# Editorial: Perinatal derivatives and the road to clinical translation, Volume II

**DOI:** 10.3389/fbioe.2022.1122728

**Published:** 2022-12-21

**Authors:** Antonietta R. Silini, Ornella Parolini, Peter Ponsaerts

**Affiliations:** ^1^ Centro di Ricerca E. Menni, Fondazione Poliambulanza- Istituto Ospedaliero, Brescia, Italy; ^2^ Department of Life Science and Public Health, Università Cattolica del Sacro Cuore, Rome, Italy; ^3^ Fondazione Policlinico Universitario “Agostino Gemelli” IRCCS, Rome, Italy; ^4^ Laboratory of Experimental Hematology, Vaccine and Infectious Disease Institute (Vaxinfectio), University of Antwerp, Antwerp, Belgium

**Keywords:** regenerative medicine, perinatal cells, placenta, secretome, preclinical (*in vivo*) studies, clinical trials, regulatory

This Research Topic issue entitled “*Perinatal Derivatives and the Road to Clinical Translation, Volume II*” builds upon Volume I to address Research Topic that are critical for moving perinatal derivatives (PnD) from preclinical lab research towards successful clinical application. These two Research Topics are a concerted effort from members of the EU-funded COST (Cooperation in Science and Technology) Action entitled “The International Network for Translating Research on Perinatal Derivatives into Therapeutic Approaches-SPRINT”. Volume II is aimed to summarize our current understandings with regard to the mechanisms and therapeutic actions of PnD, thereby 1) critically discussing basic research data that can be useful for designing clinical trials, and 2) identifying research gaps to guide future research on perinatal derivatives and streamline translation to the clinic. Finally, it will equally well put attention on current clinical trials using PnD and underline aspects that are fundamental to consider for the design of future clinical trials.

Understanding the therapeutic potential and underlying biological mechanisms is critical to allow for the identification of which PnD, either by origin, by preparation or by administration, could potentially provide the most optimal clinical benefit for a specific disease, thereby assuming that each target disease may require an optimized approach. To achieve the latter in a preclinical setting, one also needs to carefully consider—and eventually re-evaluate—the relevance and potency of classical and new *in vitro* assays to demonstrate efficacy and mode-of-action of PnD.

In the first part of this Research Topic, focusing on research and clinical studies tackling the above-described questions, Pipino et al. use an innovative in vitro model to better understand the mechanisms underlying the beneficial effects of human amniotic membrane (hAM) on diabetic foot ulcers. The applied model mimics endothelial dysfunction that occurs during *in vivo* hyperglycemia and is based on stimulating endothelial cells obtained from the umbilical cord vein of gestational diabetic mothers (GD-HUVECs) with a pro-inflammatory stimulus. Using this model, they suggest a role for the hAM to significantly reduce inflammation and to improve endothelial cell vascular network formation. Furthermore, Odet et al. contribute with two studies on the use of hAM in medication-related osteonecrosis of the jaw (MRONJ), a complication of specific pharmacological treatments such as bisphosphonates, denosumab, and angiogenesis inhibitors. In a prospective pilot study by cryopreserved hAM was applied on a compassionate use to 8 cancer patients with MRONJ. No adverse events occurred and all patients remained asymptomatic with excellent immediate significant pain relief and no infections. In a second study published by Odet et al., a pilot porcine study investigated different techniques for surgical application of hAM in an *ex vivo* model of MRONJ. Their results demonstrate that hAM had suitable mechanical properties and was easy to handle and that implantation with complete or partial coverage was the preferred choice for the MRONJ indication. This study not only once again strongly supports the use of hAM as a graft material suitable for oral surgery, but importantly, the methods described educate surgeons on the handling of hAM in oral surgery. Taking a step further towards the use of novel cell/tissue-free regenerative medicine approaches, Costa et al. analyzed whether conditioned medium or extracellular vesicles harvested from human amniotic fluid–derived stem cells (hAFSC-CM/hAFSC-EVs) could induce cardiomyocyte renewal. In this study, hAFSC were obtained from leftover samples of 2nd trimester prenatal amniocentesis (fetal hAFSC) and from clinical waste 3rd trimester amniotic fluid during scheduled C-section procedures (perinatal hAFSC), and primed under 1% O_2_ to enrich hAFSC-CM and EVs with cardioactive factors. In conclusion, this study demonstrates the promising cardiogenic effects of hAMSC-EVs and further investigations will be aimed to define their specific mechanism of action and enhance their potential translation into therapeutic opportunity. Along these lines, Dubus et al. established a protocol to obtain allogenic ECM by fully removing cell membranes and nuclei moieties from Wharton’s jelly (WJ) tissue while enhancing antibacterial properties of decellularized WJ based matrix. This study paves the way for the development of a WJ bioactive antibacterial matrix for regenerative medicine.

In the second part of this Research Topic, a review series is provided as a joint effort from the COST SPRINT Action. Being well-established that PnD act multimodally through complex mechanisms of action, in this Research Topic a four-part review series on functional assays for validation of PnD, spanning biological functions such as immunomodulation, anti-microbial and anti-cancer activities, anti-inflammation, wound healing, angiogenesis, and regeneration have been prepared by COST SPRINT consortium members. These reviews highlight fundamental points that must be considered in future research and in the development of more effective PnD therapies. Papait et al. discuss several assay relevant parameters for assessing the immunomodulatory activities of PnD with a focus on T cell and monocyte/macrophage assays. Silini et al. present the most commonly used functional assays for the assessment of antitumor and antimicrobial properties of PnD, and their advantages and disadvantages in assessing the functionality. Pozzobon et al. continue the review series by discussing the importance of potency testing in validating PnD therapeutics for regenerative medicine applications in brain, bone, skeletal muscle, heart, intestinal, liver, and lung pathologies. Finally, Flores et al. further elaborate on the beneficial modes of action of PnD on inflammation, angiogenesis and wound healing, with a special focus on vascular function as well as on cutaneous and oral wound healing.

Apart from the above-mentioned overview reviews on functional assays covering a broad spectrum of disease models and their clinical pathology, a third part of this Research Topic is devoted review manuscripts covering current knowledge regarding the application of PnD to specific diseases. Lange-Consiglio et al. provide an insightful overview of the applications of PnD in human and veterinary medicine for reproductive disorders with a focus on ovarian disorders in order to restore normal ovarian function. Administration of PnD was able to reduce oxidative stress and apoptosis of granulosa cells in injured ovarian tissue, to promote recovery of the oestrus cycle and to improve endocrine function, and thus may become new therapeutic options for infertile patients in the future. Norte-Munoz et al. performed a systematic review of published preclinical and clinical studies addressing PnD in the treatment of ocular diseases. While current clinical applications mainly focus on applications using the hAM, preclinical studies however indicate a potential for other PnD, yet underlining the need for more preclinical studies and clinical trials before PnD can be fully included in the daily ophthalmic practice. In another systematic review, Pichlsberger et al. discuss the application of PnD in wound healing, thereby highlighting the advantages and limitations of the most commonly used animal models and evaluating the impact of the type of PnD, the route of administration, and the dose of cells/secretome application in correlation with the wound healing outcome. Taken together, these systematic reviews underline how further concerted actions are needed to bridge the gap between PnD basic research, pre-clinical studies and PnD translation into the clinic for their use in various disease models and/or clinical applications. Furthermore, all these systematic reviews emphasize the importance of *in vitro* characterization of PnD and provide guidelines for this and for their preclinical use (see also Volume I of this Research Topic).

As a final part of this Research Topic, Gindraux et al. provides a comprehensive analysis of current clinical trials using PnD over various disease types. Identifying several knowledge gaps, they propose an approach for clinical trial preparation and registration in a uniform and standardized way, elaborating–using a publicly-available questionnaire—on a series of criteria that are relevant when starting a new clinical trial using PnD. This article is also important in for raising awareness to the medical field to further investigate PnD-based products in clinical practice.

Concluding, the COST SPRINT Action has here shared expertise and knowledge with the scientific community for guiding future research on PnD into streamlined translation to the clinic, thereby putting forward their therapeutic potential that will safely and surely turn into a clinical reality.

